# Burden, prevention and control of tobacco consumption in Nepal: a narrative review of existing evidence

**DOI:** 10.1093/inthealth/ihaa055

**Published:** 2020-09-11

**Authors:** Geha Nath Khanal, Resham Bahadur Khatri

**Affiliations:** Save the Children, Shambhu Marg, Sinamangal, Kathmandu, Nepal; Center for Research and Development, Surkhet, Nepal; School of Public Health, Faculty of Medicine, The University of Queensland, Brisbane, Australia

**Keywords:** challenges, Nepal, policy, prevalence, smoking

## Abstract

Tobacco consumption is one of the major public health problems in the world. Annually, 27 100 premature deaths are attributed to tobacco-related diseases in Nepal. Despite enacting different policies and strategies, the prevalence of tobacco consumption is still high. This study aims to synthesize prevalence, factors associated with its consumption and the policy initiatives for prevention and control in Nepal. This review includes peer-reviewed studies retrieved from two databases (PubMed and EMBASE) and published from 2000 to 2018, and policy initiatives on tobacco prevention and regulations in Nepal. A total of 32 studies and 5 policy documents were reviewed. Findings suggest that tobacco consumption was higher among men, illiterates, older people, people living in rural and mountainous areas and those who initiated smoking as adolescents. Peer pressure and parental/family smoking were major contributing factors for tobacco initiation. Policy analysis showed that low excise tax, weak monitoring mechanisms, poor compliance to bans on the advertisement and promotion of tobacco, smoke-free zones and insufficient programs on tobacco cessation were the major factors behind weak implementation of tobacco-control policies. Hence, targeted and high-risk group tobacco-cessation interventions, increasing taxation and strict policy implementation are crucial for effective tobacco prevention and control in Nepal.

## Introduction

Globally almost one-third of the adult population (933 million) smoke daily,^1^ which is a major risk factor for multiple non-communicable diseases (NCDs) including cancer, chronic respiratory diseases, cardiovascular diseases and diabetes.^2–4^ Tobacco consumption results in cancers in major vital organs^2^ and cumulatively causes 63% of all deaths worldwide.^5^ It is also the second leading cause of death and disability, accounting for 11.5% (6.4 million) of global deaths^1^ and 6% (148.6 million) of disability-adjusted life years.^6^ Almost 80% of these deaths occur in low- to middle-income countries where health services are limited.^7^

Annually, an estimated 27 100 deaths (14.9% of all deaths) are attributed to tobacco-related diseases in Nepal.^8^ The top three causes of death, ischemic heart disease, chronic respiratory disease and cardiovascular diseases (CVD), are associated with tobacco consumption.^8^ It is a major risk factor for lung cancer and CVD-related deaths.^8^ Treatment of tobacco-related diseases has put a burden on health services and increased the out-of-pocket (OOP) expenditure in healthcare.^9^

National surveys like the Demographic and Health Survey (DHS)^10,11^ and WHO Step-Wise Surveillance (WHO STEP) surveys^12,13^ are used to estimate the national prevalence of tobacco consumption. One-fifth of people (21.6%) were tobacco users in Nepal in 2015.^14^ Males,^4,15,16^ older people,^7,15,17,18^ the undereducated,^4,7,15,16,18^ socioeconomically poor^4,16,17^ and those living in rural^4,16,18^ and mountainous^15,18^ regions were more likely to smoke. Peer pressure^19–21^ and family environment^20,22,23^ affected early initiation.

More than 100 industries^24^ in Nepal produce different forms of tobacco products like cigarettes, bidi (rolled tobacco), hookah (nargileh), sulfa and chillum or kankad.^25^ Furthermore, smokeless tobacco (SLT) products like surti (dry tobacco leaves), khaini (lime-mixed tobacco), gutkha (areca nut) and paan (beetle quid) with tobacco ingredients are also produced in Nepal.^25^ Approximately US$90 million (4.3% of total tax) was collected as an excise in 2017, which is a lucrative contribution to national revenue.^26^

Policies regarding the prevention and control of tobacco consumption in Nepal only began in the 1990s. Taxation on international brands in 1992 and the banning of advertisements on audiovisual media in 1998 were two major interventions.^25^ Later, in 2003, the WHO Framework Convention on Tobacco Control (WHO FCTC)—the first global strategy on tobacco control―was initiated. This guided governments to formulate, implement and review comprehensive multi-sectoral national tobacco control plans and strategies. After its ratification and endorsement, Nepal endorsed legislative measures in 2010.^27^ After a decade of legal implementation, national representative surveys^7^ have reported a high and unfaltering decline in consumption prevalence. This indicates ineffective implementation of the laws with possible policy loopholes.^7^

Nepal has adopted the federal system after the constitution of 2015. The constitution provides authority to formulate, plan, implement and monitor the laws, policies and strategies related to health including tobacco prevention and control to all three tiers of government: federal, provincial (7) and local (753).^28^ Furthermore, Sustainable Development Goals (SDG) have been emphasized in the prevention and control of NCDs^29^ and tobacco prevention and control is one of the major strategies involved in it. Hence, the role of local governments will be crucial for the effective implementation of tobacco control policies.

Navigating the burden of tobacco consumption and its legal and policy measures are crucial to identify the status and implementation challenges associated with tobacco prevention and control. Therefore, this review aims to map the burden of tobacco consumption and explores the existing policy gaps associated with its prevention and control in Nepal.

## Methods

This review was conducted in two phases. First was a narrative synthesis of evidence from peer-reviewed studies focusing on prevalence and factors associated with tobacco consumption, while the review of tobacco prevention and control policies with associated implementation challenges was performed in the second phase. For narrative synthesis, two databases (PubMed and EMBASE) were used to retrieve peer-reviewed studies. Both the authors assessed full-text articles and discussed them before selection for the review process.

### Search strategy

Search terms using a combination of medical subject headings, namely, `epidemiology' OR `prevalence' OR `incidence' AND `tobacco' OR `smoking' OR `smokeless tobacco' AND `Nepal', were used to identify relevant studies. Moreover, additional relevant studies were identified by checking the reference lists of retrieved studies.

### Selection criteria

**Figure 1.**
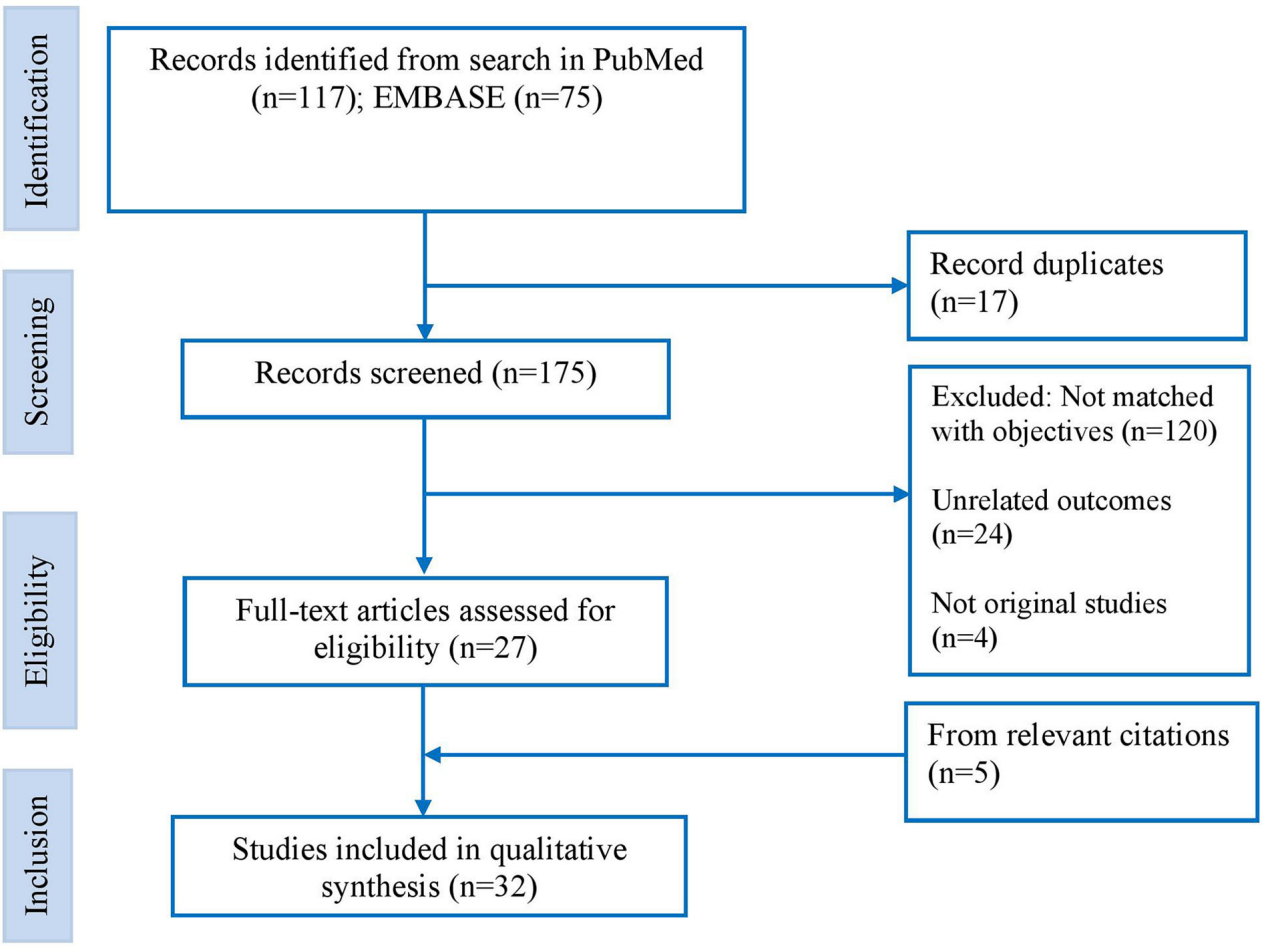
Flowchart of selection of studies.

Studies published from 2000 to 2018, written in English and conducted in Nepal met the inclusion criteria. Full-text quantitative studies that reported prevalence and/or determinants of any form of tobacco consumption were included. Studies with tobacco-related diseases like CVD, cancer and chronic respiratory diseases were excluded from the analysis (Figure 1).

### Data extraction and analysis

A table was generated that reported the first author, objective, study design, sample size and study location, the prevalence of tobacco consumption (in any form), its determinants, age of initiation (AOI) and other associated factors to extract the summary of narrative synthesis (Supplementary Table 1). Also, the existing tobacco prevention and control policies and strategies of Nepal were reviewed against current implementation challenges (Table 1).

**Table 1.** Existing tobacco regulation and control policies and their implementation challenges

MPOWER strategies	Tobacco products (prevention and control) laws	Tobacco control plan and strategies	Implementation challenges
Monitor tobacco use and prevention policies	•Provision of inspectors and their role in policy compliance^57,58^•Establishing a monitoring committee at central level to formulate and implement policies^57,58^•Delineating the roles and responsibilities of inspectors and the committee^57,58^•Using health tax fund in research and investigation of tobacco-related diseases^58^	•Developing toolkits for planning, monitoring and evaluation of and training for desk officers^60,61^•Developing tobacco control manual in line with tobacco control law^60,61^•Conducting BCC at national and subnational level^60,61^•Advocacy with concerned stakeholders•Regulation by establishing standard testing facilities^61^•Conducting compliance studies^61^•Review and amendment of legislation including violation penalty^61^	•Weak implementation of planning, monitoring and evaluation framework•Weak inspection resulting poor policy compliance•No distinct appointment criteria for inspectors (any government officers can be appointed, who has several roles beside tobacco control)•Weak stakeholder coordination mechanism•Inadequate role of the committee and their ineffective implementation•Inadequate BCC interventions at national and subnational level•Limited research conducted•Low priority of research and prevention activities from the health tax fund
Protect people from tobacco smoke	•Delineation of public places^57,58^•Designated area for tobacco consumption and its criteria^57,58^•Noticeboard targeting public, pregnant and children aged <18 y^57,58^	•Protecting from second-hand smoke•Expanding non-smoking areas^60,61^•Notifying smoke-free locations^60,61^•Awareness activities^60,61^•Conducting BCC campaigns^60,61^•Compliance monitoring by police^61^	•Poor compliance of designated roles for effective implementation•Limited smoking designated zones (only in standard hotels and some airports)•Weak regulating authorities to ensure effective implementation
Offer help to quit tobacco use	•Organize training and capacity building programs for concerned stakeholders^57^•Encourage people to quit smoking^57^	•Development of national cessation guidelines and manuals^60,61^•Establishment of tobacco cessation centers and community cessation clinics^60,61^•Establishment of quit-lines and telephone helplines^60,61^•Integration of tobacco cessation in health and education program^60,61^	•Very few health personnel have been trained on cessation•Limited numbers of counselling clinics and their low priority in tobacco cessation activities•Very few quit-line services•Integrating basic health care services with tobacco prevention and control
Warn about the dangers of tobacco	•The package and wrapper require the facts about the contains of tobacco products^58^•Warning message, symbols and graphics are to be changed regularly^57,58^•PHWs must contain at least 90% of total outer portion^59^	•Assessment of PHWs compliance•Frequent development of PHWs and its regular monitoring^60^•Coordinate with line ministries for effective legal enforcement^60,61^•Production and dissemination of BCC materials	•Handmade products have not been covered with PHWs•Covering the health warning intentionally by tobacco industries through VAT stickers^7^•Tobacco industries suiting writs and demanding judicial stay orders to procrastinate implementation•Poor coordination among concerned stakeholders
Enforce bans on TAPS	•Restriction to advertisements or promotion^57,58^•Vendors obligation to put notice board to restrict purchase/sales by pregnant and minors^57,58^•Restriction to include free or binding product sales^57,58^•Prohibition on single unit or retail sales^57^ and minimum 20 sticks cigarettes packet^58^•No government subsidies for tobacco industries	•Mobilization of local bodies, administration, civil societies and NGOs for banning TAPS^60,61^•Monitoring through coordination with other line ministries^60,61^•Conduct compliance survey and routine inspection of industries^60,61^•Development of monitoring guidelines to prohibit sales^60,61^•Ban smoking and tobacco use in public places^60,61^•Control illicit trade of tobacco products^60,61^•Registering and monitoring the tobacco shops^60^•Develop crop substitution strategy through replacement of tobacco crops^60^	•TAPS through sports by tobacco industries^56^•Ambiguity among the concerned authorities in their respective roles and responsibilities•No licensing provision to sell tobacco products•No proof is required while purchasing tobacco items to verify age or pregnancy status•Fragile implementing institutions with poor compliance•Inadequate legal provisions for registering tobacco shops•Tobacco industries violating the packaging provision by mini-packing (10 sticks in a pack)•Governmental announcement to lure the investors to revive Janakpur Cigarette Factory
Raise taxes on tobacco products	•At least 25% of excise tax has to be deposited in health fund^58^•Health fund to be used, tobacco-related research, treatment and health awareness activities^58^	•Increase resource allocation through health tax fund ^60,61^•Establish health tax fund for controlling consumption^60,61^•Assess tax structure and increase excise and tax on tobacco products regularly^60,61^	•Low excise tax without annual increment•Objection by tobacco industries/pressure groups after increasing taxation•Major portion of health tax fund is used in treatment subsidies rather that prevention, control and research activities

Abbreviations: BCC, behavioral change communication; NGOs, Non-governmental Organizations; TAPS, tobacco advertising, promotion and sponsorship; VAT, value-added tax.

### Policy documents identification

**Figure 2.**
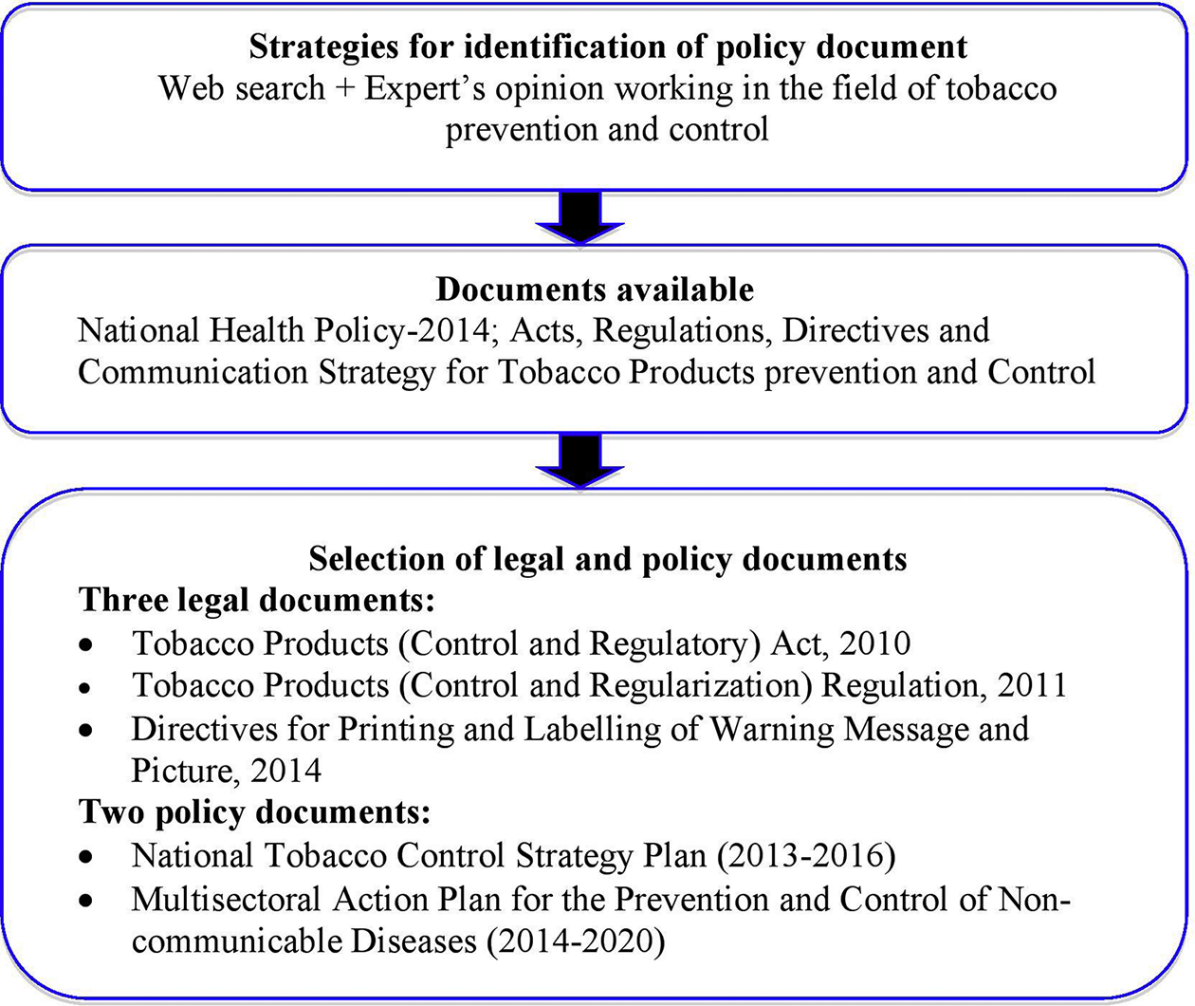
Flowchart of document review process.

The authors identified key anti-tobacco professionals by the duration of their experience working in Nepal. Policy-related documents were identified by searching websites and through personal communication with related experts. Finally, the authors decided to review three legal and two policy documents (Figure 2).

### Policy review process

**Figure 3.**
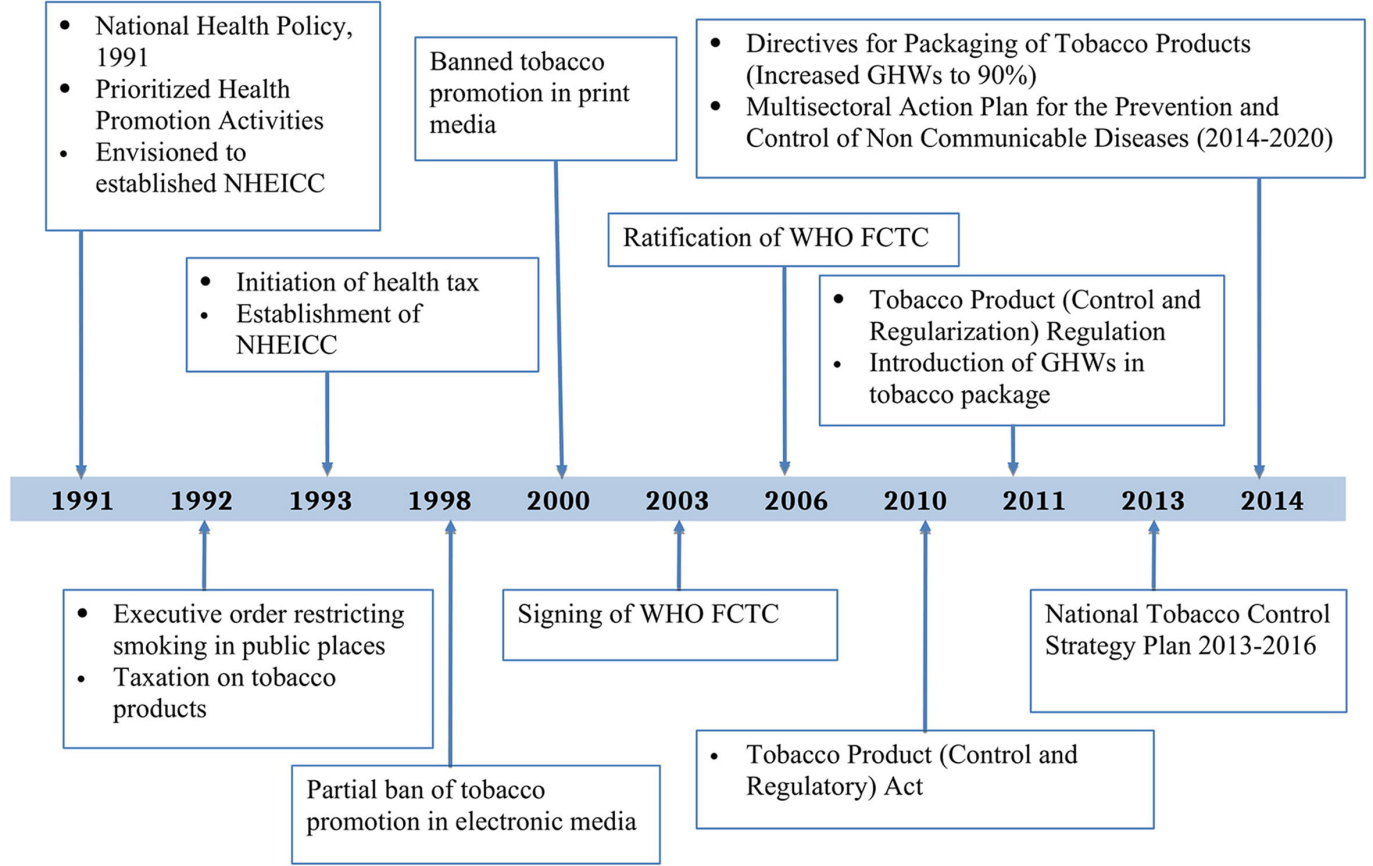
Major policy milestones on tobacco control and regulation from the 1990s in Nepal.

The chronological development of tobacco control policies after 1990 is presented in Figure 3. The Monitor tobacco use and prevention policies, Protect people from tobacco smoke, Offer help to quit tobacco use, Warn about the dangers of tobacco, Enforce bans on tobacco advertising, promotion and sponsorship, and Raise taxes on tobacco (MPOWER) strategy^30^ was used for analyzing existing tobacco prevention and control policies.

## Results

This review included 32 studies consisting of 23 primary studies and 9 secondary data analyses (Supplementary Table 1). Existing laws and policies on tobacco prevention and control with the associated implementation challenges are presented in Table 1.

The DHS, Global Youth Tobacco Survey (GYTS) and STEPS survey were the sources for secondary data analysis. Four studies were multinational while five were national surveys. Most of the studies were conducted at subnational level in cities such as Kathmandu, Pokhara and Dharan among school children or university students. Only seven studies were published from 2000 to 2010. The first authors of eight studies were foreign researchers. Seven studies reported the AOI.

### Prevalence and associated factors of tobacco consumption

The prevalence of male smokers aged 15–49 y declined from 32.5% to 27.2% while that of females decreased from 19.6% to 8.4% from 2006 to 2016.^10,31^ However, the prevalence of using any form of tobacco (smoking or SLT) in males has remained steady for a decade.^7^ The increment in consumption of SLT among males from 38.2% to 40.1% is attributed to the substitution of tobacco with SLT during the last decade.^10,31^

#### Gender

Twenty studies reported a high prevalence of smoking^4,7,15,16,19–23,32–42^ as well as consumption of SLT among males. Males were three times more likely to smoke than females.^15,22,37^

#### Age

Sixteen studies^4,7,15–18,20,22,23,32,35,36,40,43–46^ reported a significant association of tobacco consumption with increasing age. A recent study shows that males aged 45–49 y were fivefold more likely to use tobacco compared with males aged 15–19 y.^7^ Also, males aged 45–69 y were almost twofold more likely to smoke compared with males aged 15–29 y.^15^ Females aged 45–69 y were threefold more likely to smoke compared with females aged 15–29 y.^18^

#### Education

There is an inverse association of tobacco use with educational level.^4,7,15–18,42–45,47,48^ For instance, uneducated people were sixfold more likely to use tobacco and threefold more likely to chew SLT compared with those who had undergone higher education.^4^ Also, educated men were more than three times less likely to use any form of tobacco compared with their illiterate counterparts.^17^ Women with a school education were less likely to smoke compared with those without formal education.^18^

#### Marital status

The married population showed both a positive^16,17,43,44^ as well as a negative^4,46^ association with tobacco consumption. Some studies reported a higher smoking rate among divorced groups compared with married or single populations, while there were also instances where no such associations were reported.^15^

#### Socioeconomic status

Families on a low income were more likely to smoke compared with richer families.^4,7,16,17,19,44^ Also, prevalence was higher among students with better household assets,^20^ those with extravagant parents^36^ and those living in substandard accommodation.^49^

#### Place of residence

Tobacco consumption was higher among people living in rural compared with urban areas^4,16,18,44^ and those living in the hills compared with the southern plain.^15,18^ Also, males from the southern plain were more likely to use SLT compared with other regions.^7,17^

#### AOI and its factors

This review showed that tobacco consumption starts in the adolescent age groups. The mean AOI of tobacco consumption was 10.2 y among youths,^40^ 18.6 y among medical students,^50^ 15 y among rural women^43^ and 13.8 y among adolescents.^36^ The mean AOI was lower among students compared with the general population. Also, peer influences^19–23,38,40,42^ and parental smoking^19,20,22,23,47,51^ were associated with tobacco initiation. However, parental smoking did not show any such association in some other studies.^40,43,52,53^

### Policies on prevention and control of tobacco

#### Before 1990: priority to increase production

The governmental priority before 1990 was to increase the production of tobacco to fulfill national demand.^54^ For instance, Janakpur Cigarette Factory was established in 1959. Furthermore, the Tobacco Development Board was established to increase the productivity of tobacco.^54^

#### 1990–2000: prevention and control measures

Health education and promotional activities for the prevention and control of tobacco consumption were prioritized in the 1990s (Figure 3). The 1991 National Health Policy was envisioned to establish the National Health Education, Information and Communication Centre (NHEICC) under the Ministry of Health and Population. The NHEICC aimed to conduct health promotional activities, including publicizing the hazardous effects of tobacco consumption. Consequently, smoking was banned in public places in 1992.^54^ The annual budget in 1993 levied a health tax of 1 paisa (about 1/100 US cents) per stick, which was doubled in the next year. Three-quarters of this tax was used to establish a cancer hospital while the remaining funds were used for preventive and public awareness activities.^54^ Also, advertisements on both electronic (radio and television until 22:00 h)^54^ and print media were banned in 1998 and 2000, respectively.^55^

#### After 2000: reduction of demand and supply as guided by WHO-FCTC

Nepal signed the WHO-FCTC on 3 December 2003 and ratified it on 7 November 2006.^25^ This created a legal obligation to formulate and implement tobacco control plans, policy strategies and programs.^27^ As per the 1990 Treaty Act,^56^ the provisions under this convention were enforceable as Nepalese laws unless separate laws were formed. However, several suits were filed in the court after the government procrastinated in formulating separate tobacco laws as per the convention. Consequently, upon hearing the case of Parasmadi Pradhan vs The Government of Nepal, in 2007 the supreme court ordered the government to develop legislation according to the thrust of the WHO-FCTC.^57^ Despite this, tobacco laws were only endorsed after 4 y of ratification.

### Existing laws and policies on tobacco prevention and control

Existing policies and laws for tobacco prevention and control in Nepal are: (1) the Tobacco Products (Control and Regulatory) Act 2010^58^; (2) Tobacco Products (Control and Regularisation) Regulation 2011^59^; (3) a directive concerning the printing and labeling of the warning message and picture on the box, packet, wrapper, carton, parcel and packaging of tobacco products (amended in 2011)^60^ and the National Tobacco Control Strategic Plan 2013–2016^61^; and (4) the Multi-sectoral Action Plan for Prevention and Control of NCDs (2014–2020).^62^

#### Tobacco Products (Control and Regulatory) Act, 2010

The four items of the Act^58^ are price measures (demand reduction through price increment and taxation), non-price measures (protection from exposure; regulation of contents, disclosures, packaging and labeling; health education and a communication ban on tobacco advertising, promotion and sponsorship [TAPS]), demand reduction (tobacco cessation activities) and supply reduction activities (prohibiting sales to pregnant women and minors).

#### Tobacco Products (Control and Regularisation) Regulation, 2011

This regulation^59^ delineates the Act by describing the provisions and criteria of designated tobacco-consuming areas, the message for tobacco packages, package size, procedures for changing warning messages and graphics, the obligations of vendors, responsibilities of an inspector and allocation of the health fund.

#### The directive for printing, labeling and packaging of tobacco products, 2011

This directive^60^ was framed to direct tobacco industries to print and indicate clear and visible pictorial health warnings (PHWs) message and colorful pictures are different things to demonstrate the hazardous effects of tobacco consumption. After the amendment, at least 90% of the total outer portion should be covered with the picture.

#### National tobacco control strategic plan, 2013–2016

The strategic plan^61^ was formulated to strengthen the effective implementation of prevailing legislative measures. Its aims are to conduct surveillance alongside epidemiological and economic studies into tobacco control. It also includes plans to protect people from tobacco use through interventions like tobacco-cessation activities, product packaging and labeling, banning of TAPS, increasing taxation and behavioral changes. Finally, its other aims are to reduce supply by prohibiting sales to pregnant women and minors and displaying tobacco restriction signs in public places.

#### Multi-sectoral action plan for prevention and control of NCDs, 2014–2020

The plan^62^ aims to reduce preventable morbidity and premature mortality due to NCDs. It aims to reduce the relative prevalence of current tobacco use among a population aged ≥15 y by 30% (by 2025) through strengthening enforcement and compliance to existing acts and regulations.

The legal (mandatory) and policy (guiding) interventions—that are in line with the MPOWER framework—to reduce tobacco consumption and associated implementation challenges are described in Table 1.

## Discussion

This review revealed various factors behind tobacco consumption. Males were more likely to consume tobacco than females. These findings were consistent with global^1^ as well as South Asian studies.^63^ The higher prevalence of smoking among males might be due to its cultural acceptance as a masculine behavior.^37^ Furthermore, under-reporting among female consumers might be another factor.^7^ Steady prevalence among males and a declining pattern among females^7^ are inconsistent with the global trend, which shows a declining trend among both genders.^1^ This might be due to weak enforcement of tobacco laws or substitutions towards SLT. Almost one-tenth of Nepalese school children (9.4%) used tobacco, which was greater than for their Sri Lanka (9.1%) and Bangladesh (6.9%) counterparts.^40^ This might be due to inadequate interventions for tobacco prevention and control targeting the younger generations.

A higher prevalence of smoking in rural rather than urban areas^4,7,16,18,44^ might be due to limited access regarding the hazardous effects of smoking.^18^ Likewise, a higher consumption in the mountains compared with the southern plains^4,15,18^ might be due to cold weather. Furthermore, increasing the trend of substituting smoking with SLT in the southern plain might be another factor behind this.^7,17^ Different forms of SLT, like jarda, gutka, paan masala, double mazza and paan, are readily available in the southern plain. The ubiquitous and socially acceptable nature of SLT compared with cigarettes^16,17,64^ might be another factor for the soaring consumption of SLT.

The inverse relationship of education and wealth with tobacco consumption was consistent with studies from Bangladesh,^65^ Pakistan^66^ and India.^67^ Likewise, discussing the detrimental effects of tobacco among students and mothers' groups^40^ reduces its consumption. This suggests health awareness interventions are effective among high-risk groups.^40,66^ The studies from Bangladesh^68^ and India^69^ showed higher odds of tobacco consumption among widows and divorced/separated groups. However, this review did not show such an association consistently.

The consumption of tobacco starts during adolescence.^21,43,51^ This initiation is influenced by peer pressure^19–23,38,40,42^ and family environment.^19,20,22,23,47,51^ A congruent association was reported from Pakistan.^70^

### Tobacco prevention and control policies

SDG targets are to reduce the prevalence of tobacco consumption from 30.8% to 15% from 2015 to 2030.^29^ WHO FCTC and the MPOWER measures^71^ are guiding policy documents for tobacco control and prevention in Nepal. Even after a decade of robust anti-tobacco policies^26^ prevalence remains high,^7^ which indicates the frangible implementation of such policies. There are several behavioral and economic intervention challenges associated with it. Behavioral challenges are primarily warnings, monitoring and cessation programs, while taxation, marketing and the supply of tobacco products are the tobacconomics challenges.

#### Issues with taxation

Raising the tax on tobacco products is the most effective demand-reduction policy tool in tobacco control.^2,72,73^ Studies in India,^74,75^ Bangladesh^76,77^ and other Asian countries^78^ have shown price sensitivity among tobacco users. WHO recommends a 75% share of total tax in the retail price,^79^ which was just 28% in Nepal in 2014. This is one of the lowest among Asian countries.^71^ The affordability of tobacco products has not changed in the last decade, resulting in a nominal decline in tobacco consumption.^80^ Hand-made tobacco products, consumed by 14% of consumers,^13^ are beyond taxation. Furthermore, people have substituted SLT products^76,81^ after taxation increments on cigarette items. Finally, tobacco industries have increased their influence to withstand taxation policies due to their significant contribution to the national economy.

#### Implementation of bans on TAPS

Both the FCTC^79^ and Tobacco Products (Control and Regulatory) Act focus on a comprehensive ban on TAPS. This includes a restriction to advertise, promote or sponsor any tobacco product through any form of media.^58^ This further restricts transactions of tobacco products in public places^58^; however, there is weak enforcement despite the strict restrictions. Purchasing a single unit/packet is one of the drivers of cigarette smoking.^82^ Although the law prohibits selling a single unit of a cigarette, no penalties have been enforced.^7^ Instead, mini-packs (10 sticks) are available in the market these days instead of 20-stick pack.^59^

#### Interventions related to PHWs

Seventy-seven out of 181 countries have successfully implemented PHWs on tobacco products.^83^ Nepal has provision for one of the largest PHWs in the world^84^ after it increased the size from 75% to 90% in any form of packages.^60^ These warnings are frequently changed by the Ministry.^58^ Despite some good progress in the implementation of PHWs in Nepal,^84^ similar interventions in Vietnam^85^ and India^86^ were ineffective in reducing tobacco consumption.

Tobacco industries, through at least five petitions, opposed introducing PHWs on tobacco packages. Coverage of 75% PHWs was only possible after the judicial order rejecting those petitions. The court interpreted the laws and elucidated that the provision of 75% PHWs was neither unconstitutional^87^ nor against the provision of FCTC (at least 30% area coverage) as argued by tobacco industries.^24^ After this abortive attempt, the industries have started to violate the regulation indirectly by covering tobacco products' PHWs with value-added tax (VAT) stickers.^7^ Such weak legal enforcement portends the challenges of plain packaging proposed for the future.^7^

#### Promoting tobacco-cessation programs

The Act has envisioned a committee whose role—although neglected—is to provide support to quit tobacco.^58^ There are no toll-free quit-line/helpline numbers to telephone to discuss cessation.^80^ Cessation support is available in very few hospitals or clinics. Similarly, the primary healthcare system has a limited number of tobacco-cessation programs.

#### Passive smoking and smoking in public places

There are policies for creating a smoke-free environment in public places, including penalty provisions for any misdoing. However, there is poor compliance^7^ due to weak institutionalization. There is no restriction on smoking in public places like restaurants, schools/campuses or even on public transport. Multi-sectoral coordination between security, industry, finance, agriculture, health education and communication is challenging.

#### Monitoring of tobacco control, prevention and regulation policies

The STEPS, DHS and GYTS surveys^25,80^ are used to monitor prevalence at the national level. The prevalence and correlates of smoking have also been reported by other small-scaled studies. Furthermore, organizational and functional structures, which employ inspectors and committees, are not sufficiently effective in performing their designated monitoring roles.

## Implications and policy recommendations

Premature deaths from NCDs constitute more than 43.7% of all deaths in Nepal.^29^ High OOP expenditure (55%) is associated with tobacco-related diseases.^29^ Expensive treatment followed by poor health insurance coverage^29^ has further impoverished the population. Hence, reduction of consumption is the most effective strategy with which to prevent the soaring burden of NCDs and the unaffordable cost of treatment.

### Focus programs on high-risk current tobacco users

Tobacco initiation during adolescence suggests that cessation interventions should be targeted at schools. Furthermore, habitual withdrawal difficulties after getting habitual^52,88^ indicate the effectiveness of such interventions among the adolescent population. Increasing SLT consumption among males in recent years^10,31^ signifies that future interventions should be focused on SLT products.

Evidence shows that adolescents, males, the poor, the uneducated and people living in rural areas are at a higher risk and that adolescents and the peers/siblings of smokers are at a greater risk of starting smoking. Hence, it is of paramount importance to design targeted interventions such as peer education groups and to initiate discussions at schools, colleges and youth clubs. Furthermore, social media might be another way of reaching adolescents. Radio programs, street drama and quit-line support campaigns can reach all the targeted groups.

### Increase the tax on and prices of tobacco products

The key to taxation policy lies in increasing taxation, as recommended by WHO. Local governments can impose tax at the source of production. This could also bring hand-made products into the taxation system, which has not been done previously. Furthermore, periodic evaluations of the tax level and reduction of the multi-tier tax structure to a minimal tier are essential. These measures would help to minimize price variation and possible substitution towards cheaper products.^79^ Finally, the provision of a 25% fund to be used for prevention and research activities should be increased.

### Integration in routine health programs

Tobacco-cessation programs could be expanded by integrating them into routine primary healthcare.^7^ This could be accomplished through different approaches, including providing training to health workers, incorporating tobacco dependence treatment in curricula, special programs designed for the adolescent population and promoting tobacco cessation in the workplace and public places.

### Expand the jurisdiction of local government

Local governments can exercise their legal authority to formulate, implement and regulate policies, laws and guidelines related to tobacco control as provided by the Local Government Operation Act.^89^ Considering poor policy compliance, we suggest expanding the jurisdiction of local government. This could be started by devolving the authority of the 'Inspector' and the 'Committee for Control and Regulations of Tobacco Products' granted by the Act to local governments. This would ensure revenue collection, progress monitoring, compliance and an effective ban on TAPS. Furthermore, it would overcome policy implementation challenges like the ambiguity of responsibilities and poor coordination mechanisms among concerned stakeholders.

### Develop a monitoring mechanism

Strict legal implementation can bring behavioral changes. For instance, linking tobacco consumption with the paradigm of masculinity and cultural acceptance can only be minimized if existing laws are implemented effectively. This requires integrated efforts from individuals, communities, civil society and the local government. Effective policy compliance mechanisms, namely, banning consumption and sale around public places, enforcing the legal age and non-pregnancy status for purchasing, shops’ licensing, prohibiting single-unit sales/purchases, intentional flouting of the law by tobacco industries and attracting consumers through mini-packs, should be monitored effectively.

Also, almost one-eighth of tobacco users^13^ consume homemade products like hand-rolled cigarettes, tobacco pipes, cigars, cheroots, cigarillos and shisha. These consumers are neither affected by taxation nor by PHWs. Thus we suggest a regulatory mechanism at the local level. This mechanism can restrict production and consumption as well as ensuring the effective implementation of policies.

There are some limitations to this study. It provides a narrative summary of the evidence. Quality grading was not possible, rendering a meta-analysis unfeasible. Furthermore, the subtle definition of tobacco consumption was quite difficult. Some defined it as those who had smoked in the past 30 d^15,18^ while others have not imposed such a time limitation.^4,7,16,17,44^

### Conclusion

Higher prevalence of tobacco consumption among males, the illiterate, the poor, older people and those living in a rural area illuminates the target groups that need to be targeted when designing and implementing tobacco control interventions. The influence of peer pressure and family environment in tobacco initiation indicates that tobacco prevention activities should start in schools. Nepalese tobacco-control policies are in line with WHO-FCTC and MPOWER strategies; however, there are various associated implementation challenges. Increasing the tax base and amount, effective implementation of a ban on TAPS, counseling and support for tobacco cessation are equally important along with PHWs. The federal structure and expansion of the jurisdiction of local government could be windows of opportunity to address current weak policy and regulation compliance.

## Supplementary Material

ihaa055_Supplemental_File
