# Supplementary material for: Burden, prevention and control of tobacco consumption in Nepal: a narrative review of existing evidence
Source: Int Health. 2020 Sep 11;13(2):110–21. doi: 10.1093/inthealth/ihaa055 (PMC7902273; doi:10.1093/inthealth/ihaa055)
Supplement: ihaa055_Supplemental_File [file ihaa055_supplemental_file.docx]

***Table 1:*** *Summary of studies on prevalence and factors associated with tobacco consumption in Nepal*

| ***Study*** | ***Objective*** | ***Study Design*** | ***Sample size*** | ***Areas of study*** | ***Prevalence*** | | | ***Factors associated with consumption*** | | ***Age of initiation and associated factors*** |
| --- | --- | --- | --- | --- | --- | --- | --- | --- | --- | --- |
|  |  |  |  |  | ***Male; % (95%CI)*** | ***Female % (95%CI)*** | ***Overall% (95%CI)*** | ***Increase if*** | ***Decrease if*** |  |
| Jha et al., 2002^21^ | To estimate prevalence among college students | Cross-sectional | 468 college students aged 16-26 years | Dharan, Sunsari | 32.9 | 3.1 | 24.8,  *12.2  **4.1 | Male, peer pressure | NA | NA |
| Niraula., 2004^46^ | To estimate prevalence, patterns and factors | Cross-sectional | 2,349 females aged 15 years and above | Dharan, Sunsari | NA | 12.9 (7.2% for 15-49 years &  37.7% for 50 years and above) | NA | Increased age | NA | NA |
| Sreeramareddy et al., 2008^20^ | To estimate the prevalence and correlates | Cross-sectional | 1,590 students aged 17-32 years | Pokhara, Kaski | *14 [11.8-16.1]  **9.3 [7.5-11.1] | *1.9 [0.8-2.9]  **1.9 [0.8-2.9] | *9.4[8.0-10.9]  **6.5 [5.3-7.8] | Increased age,  Male | Fewer household assets | Friends, family members, tobacco purchasing |
| Sreeramareddy et al., 2010^34^ | To determine the current use and the perception towards it | Cross-sectional | 152 medical students | Malaysia, India, Pakistan, Bangladesh and Nepal | 22.8 | 8.2 | 17.8 | Male | NA | NA |
| Binu et al., 2010^22^ | Prevalence and correlates of smoking | Cross-sectional | 816 college students | Pokhara, Kaski | 48 | 18 | 17 | Male, tobacco chewers, peer pressure, smoker family members | NA | NA |
| Aryal et al., 2011^33^ | To determine Prevalence and perceived risk of cigarette smoking | Cross-sectional | 340 public health students | Kathmandu valley | NA | NA | 16 | Male | NA | NA |
| Ghimire et al., 2013^51^ | Explore the magnitude and causes of smoking | Cross-sectional | 345 medical and male dental students | Dharan, Sunsari | 38.4 | NA | NA | Parental smoking, use of other drugs | NA | 28.4% initiated before 15 years |
| Adhikari et al., 2014^32^ | Assess the gender-wise difference in prevalence of risk factors of NCDs | Cross-sectional | 1,240 respondents aged 15-64 years | 6 districts of CDR | 42.1 | 18.4 | 31.1 | Current tobacco user, frequent tobacco users,  Increased age | NA | NA |
| Sreeramareddy et al., 2014^16^ | Estimate the prevalence and social determinants of smoking and SLT | Cross-sectional | 4,121 men and 12,674 females aged 15-49 years | India, Pakistan, Maldives, Indonesia, Cambodia, Philippines, Timor Liste, Nepal | *33.6 [31.2-36]  **34.8 [32.4-37.2] | *9.7[8.7-10.8]  **4.7 [3.8-5.7] | NA | Increased age, married, rural | Higher education, higher economic status | NA |
| Sreeramareddy et al., 2011^4^ | Estimates the prevalence, distribution, and correlates of tobacco use | Cross-sectional | 15,190 population between 15-59 years | Nation-wide study using NDHS 2006 | *32.8 [30.6-35.2]  **38 [35.8-40.3]  ***56.5 [54.1-58.8] | *15.8 [14.5-17.15  **5.0 [4.3-5.8%]  ***19.6[18.3-21.0] | *20.7 [19.5-22.0]  **14.6 [13.5-15.5]  ***30.3 [28.9-31.7] | Increased age, poorest,  rural, male, divorced/ separated | Higher education, Southern plain | NA |
| Sinha et al., 2015^35^ | Estimate the trends of SLT use among adults aged 15-49 years | Cross-sectional | NA | Bangladesh, India and Nepal | ** 31.6[27.6-35.8] | ** 4.7 [3.6-6.0] | ** 17.5 [15.5-19.7] | Male, increased age | NA | NA |
| Khanal et al., 2013^17^ | Estimates the prevalence and determinants of tobacco use among males | Cross-sectional | 4,121 males aged 15-49 years | Nationwide study using NDHS 2011 | * 33.6 [31.2-36]  ** 34.8 [32.4-37.2]  *** 51.9[49.6-54.3] | NA | NA | Increased age, married/defacto, manual workers, southern plain, | Higher education,  higher economy  CDR, Television | NA |
| Sinha et al., 2016^44^ | To estimate the age-standardized prevalence and correlates of tobacco use | Cross-sectional | NA | Multi-national study of 6 countries | #55.6 [53.4-57.8]  ##17.9 [16.0-20.1] | #15.7 [14.2-17.3]  ##1.45 [10.9-1.94] | NA | Increased age,  Married | Higher education, Rich,  Islam religion | NA |
| Bista et al., 2015^18^ | To understand the socio-economic determinants of tobacco use | Cross-sectional | 2,797 women aged 15-69 years | Nation-wide (STEPS survey 2013) | NA | *10.3 [8.8-11.9]  ** 4.8 [3.8-6.0]  #14.1 [12.4-15.9] | NA | Increased age,  Rural | Southern plain,  Higher education, | NA |
| Shakya et al., 2015^37^ | To understand the gender difference in risk factors for NCDs | Cross-sectional | 191 medical students aged 20-25 years | Kathmandu, Valley | *25.2 | *8.3 | *18.8 | Male | NA | NA |
| Aryal et al., 2015^23^ | Prevalence and correlates of smoking susceptibility and intention to smoke | Cross-sectional | 2,878 school adolescents | 31 districts (second GYTS) | NA | NA | Smoking susceptibility: 22.8% [21.2-24.5]  Intention to smoke: 11.4% [10.2-12.6] | Increased age,  Male, Offered free cigarette | Taught in curriculum | Parental use, Friend use, |
| Kabir et al., 2013^40^ | To compare the tobacco consumption among youths | Cross-sectional | 1,444 school children aged 13-15 years | Bangladesh, Nepal, and Sri-Lanka | *13.2 | *5.3 | *9.4 | Increased age, male, peer using tobacco use | School curriculum, the discussion in the classroom | AOI: 10.24 years |
| Ayral et al., 2015^41^ | To assess the perceived risk and benefit of smoking | Cross-sectional | 315 students aged 18-24 | Kathmandu | *28.4 [21.1-35.6] | *5.4 [1.9-3.6] | *16.2 [12.3-31.5] | Male | NA | AOI:  Male: 16.6[15.8-17.3]  Female: 17.7[15.8-19.4] |
| Mishra et al., 2015^50^ | Assess the prevalence of modifiable risk factors of NCDs | Cross-sectional | 191 medical students aged 20-25 years | Kathmandu | *25.2 [17.7-34.0]  ** 5.0 [1.9-10.7]] | *8.3 [3.1-17.3]  **0 | *18.8 [13.6-25.1]  ** 3.1 [1.2-6.7] | NA | NA | AOI: 18.6 [17.4-19.8] |
| Aryal et al., 2015^15^ | To assess the distribution and determinants of NCDs risk factor | Cross-sectional | 4,143 people aged 15-69 years | Nationwide (STEPS Survey 2013) | *27 [23.8-30.5] | ** 10.3 [8.8-12.0] | *18.5 [16.6-20.6] | Increased age, Male | Higher education,  Southern plain | NA |
| Bhaskar et al., 2016^19^ | Prevalence and correlates of ever use of tobacco of any form | Cross-sectional | 1,540 students aged 10-18 years | Bara | 31 | 14.4 | 25.3 | Male, Ethnic minorities  Family members using tobacco, Peer pressure,  Exposure at home,  Exposure at public places | Knowledge about the harmful effect | AOI: 13.4 years |
| Khatri et al., 2015^43^ | Prevalence and determinants of tobacco use | Cross-sectional | 110 women aged 15-49 years | Dailekh | NA | 43.6 | NA | Increased age, Education, Agriculture, Married | NA | AOI: 15 years  Peer pressure. |
| Pradhan et al., 2016^52^ | Prevalence of smoking, unsuccessful quitting attempts and associated factors | Cross-sectional | 1,312 students aged 14-19 years | Dharan, Sunsari |  |  | 13.7 | NA | NA | NA |
| Pradhan et al., 2013^36^ | Estimate the prevalence and correlates of ever tobacco use | Cross-sectional | 1,312 students aged 14-19 years | Dharan, Sunsari | 33.6 [30.2-36.9] | 4[2.6-5.3] | 19.7 [17.7 -21.6] | Increased age, Male, Joint family, Receiving more pocket money, Indigenous population | Studying in private school | AOI: 13.79 years |
| Oli et al., 2013^49^ | Estimate the prevalence of risk factors of NCDs in slum areas | Cross-sectional | 689 slum households | Kathmandu | *51.9  **38.5 | *24.1  **15.3 | *35.6  **24.8 | NA | NA | NA |
| Pokhrel et al., 2006^38^ | Estimate the prevalence and correlates of tobacco use | Cross-sectional | 377 health professionals | Dhading and Ilam | 32.4 | 3.2 | NA | Alcohol users, Male,  Peer groups |  | NA |
| Singh et al., 2017^48^ | Explore the factors associated with tobacco consumption | Cross-sectional | 426 pregnant women | Dhanusha | NA | # 21.4  * 13.4 | NA | Age between 20-34 years, ethnic minorities, smoking,  spousal violence, non-vegetarian, alcohol users | Education, exposure to mass media, attending mothers’ group meeting | NA |
| Sah et al., 2016^42^ | Estimate the prevalence and factors associated with tobacco use | Cross-sectional | 205 residences | Dhankuta | 67 | 47.1 | 57.1 | Male, poor, peer pressure | Higher education | NA |
| Pradhan et al., 2015^53^ | Explore the prevalence of tobacco use and associated factors among female adolescent students | Cross-sectional | 618 students | Dharan, Sunsari | NA | 2.4 [1.2 -3.5] | NA | Alcohol | NA | AOI: 13.6 years |
| Barakoti et al., 2017^47^ | To explore the prevalence and associated risk factors of tobacco use during pregnancy | Cross-sectional | 436 women aged 15-49 years | Sankhuwasabha | NA | 17.2 [13.7-20.7] | NA | Agriculture, more siblings,  alcohol, tobacco users in the family | Higher education | NA |
| Karmacharya et al., 2017^39^ | Estimate the prevalence of smoking and oral tobacco | Cross-sectional | 1,073 adults | Kavre | *35.6  **12.3 | *14.2  **0.5 | *23.1  **5.4 | Male, Ethnic majorities,  Alcohol consumers | NA | NA |
| Shrestha et al., 2019^7^ | To examine prevalence and to explore the determinants of tobacco use | Cross-sectional | 4,063 males and 12,862 females aged 15–49-years | Nationwide (NDHS 2016) | *27.4  **40.1  # 52.3 | *5.5  **3.8  #8.4 | NA | Increased age, male, poor | Higher education | NA |

**: Smoking; **: Smokeless tobacco; ***: both; ^#:^ Tobacco of any form; ^##:^ Tobacco of both (Smoking and SLT) forms, NA: not available/applicable; CI: confidence interval; CDR: Central Development Region: AOI: Age of initiation*
